# Muscle MRI as a Useful Biomarker in Hereditary Transthyretin Amyloidosis: A Pilot Study

**DOI:** 10.3390/genes12111786

**Published:** 2021-11-11

**Authors:** Guido Primiano, Tommaso Verdolotti, Gabriella D’Apolito, Andrea Di Paolantonio, Valeria Guglielmino, Angela Romano, Gabriele Lucioli, Marco Luigetti, Serenella Servidei

**Affiliations:** 1 Fondazione Policlinico Universitario A. Gemelli IRCCS, 00168 Rome, Italy; guidoalessandro.primiano@policlinicogemelli.it (G.P.); tommaso.verdolotti@policlinicogemelli.it (T.V.); gabriella.dapolito@policlinicogemelli.it (G.D.); guglielmino.valeria@gmail.com (V.G.); lucioli.gabriele@gmail.com (G.L.); serenella.servidei@unicatt.it (S.S.); 2 Università Cattolica del Sacro Cuore, 00168 Rome, Italy; andrea.dp1988@gmail.com (A.D.P.); angela.romano12@gmail.com (A.R.)

**Keywords:** *TTR*, amyloid, muscle MR, biomarker, progression

## Abstract

Hereditary transthyretin amyloidosis (ATTRv, v for variant) is a severe and heterogeneous multisystem condition with a prevalent peripheral nervous system impairment, due to mutations in the transthyretin gene. Considering the introduction of different disease-modifying therapies in the last few years, a need of reliable biomarkers is emerging. In this study, we evaluated muscle MRI in a cohort of ATTRv patients in order to establish if the severity of muscle involvement correlated with disease severity. Linear regression analysis showed a significant positive correlation between the total fatty infiltration score and NIS, NIS-LL, and Norfolk, and an inverse correlation with Sudoscan registered from feet. In conclusion, we demonstrated the role of muscle MRI in ATTRv as possible disease biomarker, both for diagnostic purposes and for assessing the severity of the disease.

## 1. Introduction

Hereditary transthyretin amyloidosis (ATTRv, v for variant) is a severe and heterogeneous multisystem condition with a prevalent peripheral nervous system impairment, due to mutations in the transthyretin (*TTR*) gene [1,2]. ATTRv amyloidosis represents a diagnostic challenge considering the great variability in clinical presentation and multiorgan involvement [1,2]. Generally, this neurogenetic disease is characterized by polyneuropathy, but clinicians should consider also the frequent cardiac, ocular, and gastro-intestinal impairment [3,4,5]. However, the pattern of clinical impairment may vary according to the geographic area. In endemic areas, namely Portugal, patients present early-onset (third to fourth decade) ATTRv and deteriorate quickly because of autonomic dysfunction and rapid progression of the sensory-motor deficit [6]. Conversely, late-onset ATTRv, characterized by a slowly progressive polyneuropathy (affecting predominantly the large nerve fibres), often with cardiac involvement but with less autonomic dysfunction, is peculiar of non-endemic areas [7].

A diagnosis of ATTRv amyloidosis must be confirmed by *TTR* gene sequencing to detect amyloidogenic variants [1,2]. Once diagnosis has been established, we evaluate in clinical practice the extent of ATTRv patients disability with useful tools, including the Familial Amyloid Polyneuropathy (FAP) staging system, the Neuropathy Impairment Score (NIS), a clinical compound score based on examination of muscle weakness, sensory loss and stretch reflexes in the limbs, and its subset, the NIS-lower limbs (NIS-LL) score, and the Norfolk Quality of Life-Diabetic Neuropathy (QoL-DN) questionnaire to estimate quality of life [1,2].

The last few years have been characterized by a significant change in disease management due to a deeper knowledge of the phenotype-genotype correlations, but above all for the introduction of disease-modifying therapies (DMT) [8]. There is therefore a clear need for reliable disease biomarkers, both able to monitor the efficacy of pharmacological treatment or the progression of the disease but also to early identify when asymptomatic carriers of pathogenic variants in the *TTR* gene manifest the disease.

In the context of neuromuscular disorders, muscle MRI has proven to be a technique that can be used both as a diagnostic tool, as many hereditary myopathies have a specific muscle involvement (“pattern”), and as a tool to evaluate the progression of pathology [9,10]. Here, we present the first cohort of ATTRv patients in whom pelvic and lower limbs muscle MRI was evaluated in order to assess a possible diagnostic pattern and to establish if the severity of muscle involvement, graded with specific scoring, was a biomarker of disease severity.

## 2. Materials and Methods

We prospectively enrolled patients affected by ATTRv admitted to our Neurology Department. As a control group, we evaluated age and sex-matched healthy subjects with non-specific myalgia but without clinical and laboratory evidence of neuromuscular pathologies. All subjects underwent complete general and neurological examination. Detailed demographic, clinical, genetic and neurophysiological data were collected for each patient: type of *TTR* mutation, gender, age, FAP stage, Sudoscan values, NIS and its sub-score NIS-LL, and Norfolk QoL-DN. At the time of the clinical examination all subjects underwent pelvis and lower limb MRI according to the previously described protocol [9]. In particular, scans were independently evaluated by two investigators with experience in muscle imaging blind to clinical data. Twenty-seven muscles were evaluated at the pelvis, thigh and calf levels using a 5-point scale [10]. Stage 0 is referred to a normal appearance; stage 1 (mild) to traces of increased signal intensity on the T1-weighted MRI sequences; stage 2 (moderate) to increased signal intensity (MRI) with beginning confluence in less than 50% of the muscle; stage 3 (severe) to increased signal intensity (MRI) in more than 50% of an examined muscle; and stage 4 (end-stage disease) to a state when the entire muscle is replaced by increased signal intensity (MRI) [10]. We finally calculated the “total fatty infiltration score” resulting from the sum of pelvis, tight and leg muscles scoring. STIR sequences were performed to evaluate the presence or absence of signal hyperintensity in each muscle. Asymmetric muscle involvement was defined as a side to-side score difference of at least one point. Statistical analysis was performed with “SPSS 22” (“Statistical Package for Social Science”). The significance level was set at *p* < 0.05.

The study was approved by the Ethics Committee of the Università Cattolica del Sacro Cuore (Rome, Italy; ID 1493) and all participants signed an informed consent prior to inclusion. All procedures were conducted in compliance to the ethical standards laid down in the 1964 Declaration of Helsinki and its later amendments.

## 3. Results

Ten patients, 1 female and 9 males, were enrolled in this study. Mean age at onset was 63.2 ± 6.4 years (range 50–72). Mean age at examination was 67.4 ± 6.4 years (range 53–74). Clinical and genetic data are summarized in Table 1.

**Figure 1:**
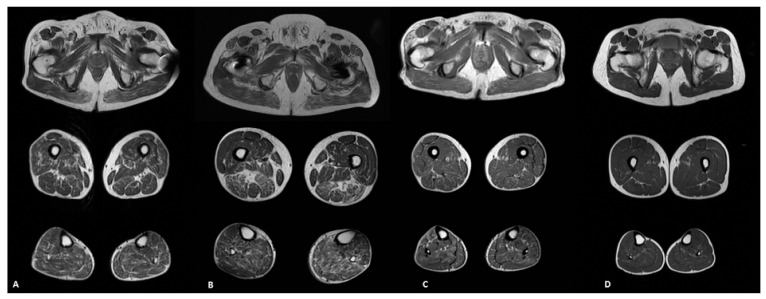
Muscle MRI pattern of fatty infiltration in ATTRv patients (#2 in (**A**); #6 in (**B**); #1 in (**C**)) and in a healthy subject (**D**). Tensor fascia latae and soleus were the most frequently involved muscles, with relative sparing of the iliopsoas, rectus femoris, gracilis and anterior tibilias muscles (**A**,**B**); in only one case the anterior tibialis presented an asymmetric severe adipose substitution (**C**). No fatty replacement was found in healthy subject (**D**).

None of the 10 patients had normal muscle MRI. Tensor fascia latae and soleus were the most severely involved muscles with a score of 2 or higher in 90% of the patients (9/10), with relative sparing (score ≤ 1) of the iliopsoas, rectus femoris, gracilis and muscles of the anterior compartment of the leg (Figure 1A–C).

Considering pelvic muscles, we identified a total fatty infiltration score ranging from 12 to 36 (mean 26.3 ± 10.1 SD). In particular, the most affected muscles were glutei (gluteus maximus: mean 3.6 ± 0.8 SD; gluteus medius: average 2.8 ± 1 SD; gluteus minimus: medium 2.9 ± 1.4 SD). Iliopsoas was relatively spared (score ≤ 1 in 10/10).

Considering thigh muscles, we observed a total fatty infiltration score between 20 and 45 (average 31.2 ± 6.3 SD). Tensor fasciae latae muscle and semimembranosus were the most frequently involved muscles with a significant degree of muscle degeneration (score ≥ 2 respectively in 9/10 and 8/10 patients; total mean 4.3 ± 1.3 SD and 3.9 ± 0.9 SD respectively). Instead, the rectus femoris and gracilis were the least affected muscles (score ≤ 1 respectively in 8/10 and 9/10 patients; total mean 2.4 ± 0.8 SD and 2 ± 0.9 SD respectively).

Considering leg muscles, we observed a total fatty infiltration score between 23 and 39 (mean 28.2 ± 5.5 SD). The most involved muscles were soleus (mean 3.9 ± 0.9 SD), the medial gastrocnemius (mean 3.4 ± 1.3 SD) and lateral gastrocnemius (mean 3.3 ± 1.2 SD), with a relative sparing of the muscles of the antero-lateral compartment.

**Figure 2:**
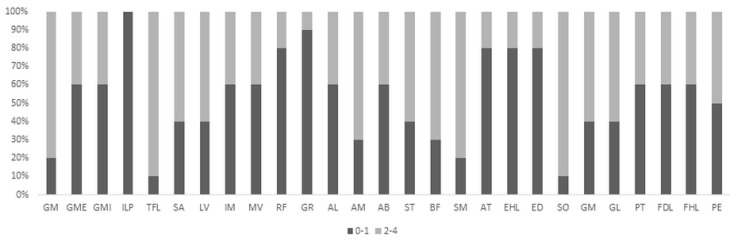
Pelvic and lower limb muscle involvement in ATTRv patients. Black bars indicate affected muscles with score 0–1; grey bars indicate affected muscles with score 2–4. GM gluteus maximus, GME gluteus medius, GMI gluteus minimus, ILP iliopsoas, TFL tensor fascia latae, SA sartorius, LV lateral vastus, IM intermedius vastus, MV medial vastus, RF rectus femoris, GR gracilis, AL adductor longus, AM adductor magnus, AB adductor brevis, ST semitendinosus, BF biceps femoris, SM semimembranosus, AT anterior tibialis, EHL extensor hallucis longus, ED extensor digitorum, SO soleus, GM gastrocnemius medial, GL gastrocnemius lateral, PT posterior tibialis, FDL flexor digitorum longus, FHL flexor hallucis longus, PE peroneus.

Detailed involvement of each muscle is represented in Figure 2.

Linear regression analysis showed a significant positive correlation between total fatty score and NIS (Spearman’s rho = 0.802, *p* = 0.005), NIS-LL (rho = 0.821, *p* = 0.004), and Norkfolk QoL questionnaire (rho = 0.742, *p* = 0.014). An inverse correlation was found between the fatty infiltration score and Sudoscan data from feet (rho = −0.638, *p* = 0.047).

**Figure 3:**
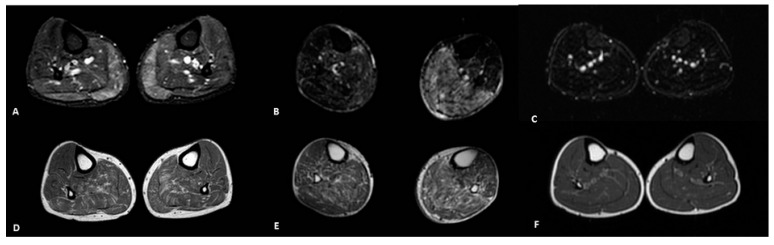
Muscle disease activity in ATTRv patients (#3 in (**A**,**D**); #6 in (**B**,**E**)) and a control subject (**C**,**F**). STIR hyperintensities in gastrocnemii muscles without fatty degeneration as a sign of early involvement (**A**,**D**); STIR hyperintensities in posterior compartment of the leg with a severe muscle involvement (**B**,**E**); no STIR abnormalities in the control subject (**C**,**F**).

Abnormalities of the STIR sequences in at least 1 muscle were present in 80% of patients (8 of 10), and in 15.5% of the examined muscles (Figure 3A,D,B,E). The muscles that most frequently showed an alteration in STIR, not always associated with a fatty replacement, were biceps femoris (65%), vastus intermedius (50%), vastus medialis (50%) and gastrocnemius medialis (65%).

Asymmetric involvement was detected in 70% of patients, with a number of involved muscles ranging from one to six.

As a control group, we included 10 age-matched healthy subject (mean age 66.8 ± 5.7 years, range 51–73). In the control group, muscle MRI documented an absence of fatty replacement or minor physiological degenerative changes (score ≤ 1 in 10/10 subjects; total fatty infiltration ranged from 0 to 16; Figure 1D and Figure 3C,F). No STIR abnormalities were detected in these subjects. NIS and NIS-LL scales were also unremarkable with the exception of distal reflexes loss in some subject (range 0–8). In the control group, we did not find any significant correlation.

## 4. Discussion

ATTRv amyloidosis is a rare neurogenetic disorder characterized by a predominant involvement of the peripheral nervous system, but typically affecting further organs such as heart, kidney and eye [1,2]). The clinical heterogeneity and the presence of DMT justify the need for reliable biomarkers able to promptly direct the clinician towards an early diagnosis, to monitor the disease progression and to document the effectiveness of a specific treatment.

Over the past few years, numerous papers showed the key role of muscle MRI in neuromuscular diseases as imaging biomarker [11]. In particular, in some inherited muscular pathologies, the pattern of muscle involvement may contribute to address the specific molecular diagnosis [12,13,14,15,16,17]. The characteristic multisystem involvement and, consequently, the presence of non-neurological manifestations in ATTRv, may lead to a significant delay in diagnosis. Similarly, even when the main symptoms are neuropathic, misdiagnosis can be made due to the variety of initial clinical and neurophysiological findings, potentially mimicking other peripheral neuropathies. From this point of view, the pattern of muscle involvement could be among the criteria useful for suspecting ATTRv and allowing a more rapid diagnosis. In particular, the involvement of tensor fascia latae and soleus muscles associated with a relative sparing of iliopsoas, gracilis and the muscle of the anterolateral compartment of the leg can be a helpful diagnostic hint, orienting the genetic investigations, especially in patients with concomitant clinical findings such as neuropathy or cardiac, eye and kidney involvement.

Compared to what was described in patients affected by spinal muscular atrophy (SMA) or other inherited neuropathies, our patient cohort presented similarities and differences [9,18]. As previously reported in subjects classified as type 3 ambulant SMA, in our cohort the glutei, the gastrocnemii and the soleus were also significantly involved. However, differently from what was described by Brogna and colleagues in SMA 3, patients affected by ATTRv presented frequently severe fatty degeneration of the tensor fasciae latae and semimembranosus muscles with a less involvement of the gracilis, quadriceps muscles and the flexor and extensor compartments of the leg. Furthermore, the muscle imaging pattern was also different from what is seen in patients with Charcot–Marie–Tooth disease type (CMT) 1A characterized by the predominant involvement of the anterolateral compartment of the leg [18]. Vice versa, muscle impairment may be similar in patients with mutations in *MFN2* (CMT2A) for the predominant involvement of the posterior compartment; unfortunately, the involvement of the pelvic and thigh muscles was not previously assessed in this disorder [18].

Considering the positive correlation observed between the total fatty score and clinical scales and the inverse correlation with Sudoscan data from feet, commonly used in evaluating the ATTRv progression [19], muscle MRI can be considered an imaging biomarker of disease severity and, in perspective, capable of quantify disease progression and response to drug treatments. The latter aspect is even more relevant for the actual possibility for many patients to have access to DMT.

Finally, we can speculate about a possible role of muscle MRI in monitoring pre-symptomatic carriers. In fact, different criteria to define ATTRv onset, during pre-symptomatic subject monitoring, have been proposed [20]. Considering instrumental tools, the investigation proposed in the past years [20] have been helped by new ones [4,21,22]. Muscle MRI could represent a new tool in this setting, detecting early signs of ATTRv amyloidosis, in order to initiate DMT. Regarding this specific aspect, STIR hyperintensities, an early sign of muscle involvement, could be considered a first manifestation of the disease in clinically non-symptomatic patients as we have identified STIR hyperintensities also in muscles without fatty degeneration.

In conclusion we documented a characteristic muscle involvement by MRI in patients with ATTRv, and we propose it could be considered as possible biomarker both for diagnostic purposes and for assessing the severity of the disease. Our data suggest a future role of this technique in clinical trials for monitoring response to therapy.

## Figures and Tables

**Table 1:** Demographic and clinical findings of ATTRV patients.

Patient and Sex	Mutation	Age at Onset	Age at Examination	Disease Duration (Months)	FAP Stage	NIS Scale	NIS-LL Sub-Scale	Norfolk QoL-DN	Total Fatty Infiltration Score	BMI	CK
#1, M	Phe64Leu	64	73	104	1	21	17	68	70	23	151
#2, M	Phe64Leu	72	74	24	1	97.5	52	84	100	24.3	104
#3, M	Val30Met	59	66	84	2	77.7	48.7	78	80	24.7	338
#4, M	Val30Met	66	69	30	1	60	45	56	94	23.7	212
#5, M	Val30Met	70	70	8	1	13	7	1	58	24.7	98
#6, M	Ile88Leu	62	65	34	2	55	30	54	94	32.8	162
#7, M	Phe64Leu	50	53	32	1	28.5	8	22	67	29.3	984
#8, M	Val30Met	66	69	30	1	31.5	27.5	21	59	23.9	156
#9, M	Phe64Leu	58	62	48	1	50	33	36	91	29.1	127
#10, F	Val30Met	65	73	91	1	18	16	51	79	26	115

***Legend to the Table:*** FAP, Familial Amyloid Polyneuropathy; NIS, Neuropathy Impairment Score; NIS-LL, lower limb Neuropathy Impairment Score; Norkfolk QoL-DN, Norfolk Quality of Life-Diabetic Neuropathy; BMI, body mass index, CK, creatin kinase.

## Data Availability

The data presented in this study are available on request from the corresponding author.
